# An interpretable deep learning approach for designing nanoporous silicon nitride membranes with tunable mechanical properties

**DOI:** 10.1038/s41524-023-01037-0

**Published:** 2023-05-27

**Authors:** Ali K. Shargh, Niaz Abdolrahim

**Affiliations:** 1 Department of Mechanical Engineering, University of Rochester, Rochester, NY 14627 USA; 2 Materials Science program, University of Rochester, Rochester, NY 14627 USA; 3 Laboratory for Laser Energetics, University of Rochester, Rochester, NY 14627 USA

**Keywords:** Computational methods, Nanoscale materials

## Abstract

The high permeability and strong selectivity of nanoporous silicon nitride (NPN) membranes make them attractive in a broad range of applications. Despite their growing use, the strength of NPN membranes needs to be improved for further extending their biomedical applications. In this work, we implement a deep learning framework to design NPN membranes with improved or prescribed strength values. We examine the predictions of our framework using physics-based simulations. Our results confirm that the proposed framework is not only able to predict the strength of NPN membranes with a wide range of microstructures, but also can design NPN membranes with prescribed or improved strength. Our simulations further demonstrate that the microstructural heterogeneity that our framework suggests for the optimized design, lowers the stress concentration around the pores and leads to the strength improvement of NPN membranes as compared to conventional membranes with homogenous microstructures.

## Introduction

**N**ano**p**orous silicon **n**itride (NPN) membranes are freestanding ultrathin films that offer outstanding combination of high permeability and strong selectivity based on molecular size or charge^1–3^. Such properties make NPN membranes attractive in a broad range of applications including rapid detection of intact SARS-CoV-2, biological separations, DNA translocation, and miniaturization of hemodialysis^4–6^. Despite their growing use, it is well-established that for practical application of NPN membranes in some of the fields, their mechanical properties must be carefully tuned. For instance, high strength is needed for successful application of NPN membranes in wearable hemodialysis devices, while enhanced deformability is desired for high-throughput applications. The mechanical properties of NPN membranes are highly dependent on their microstructure. As a result, the first step in designing NPN membranes with desired mechanical properties is to understand the relationships between the microstructural descriptors of the NPN membranes such as: pore shape, pore pattern, and pore density with their mechanical properties. In practice, it is excessively easier to conduct such forward design approach through physics-based simulations including molecular dynamics (MD) or finite element (FE) simulations in comparison with the experiments^7–12^. In our earlier works^13–15^, we used MD simulations to investigate the mechanical behavior of NPN membranes with different microstructures. We revealed that the deformability of the NPN membranes can be significantly improved using hexagonal pore patterns with same-size circular pores. Our simulations further demonstrated that the amorphous microstructure provides better opportunity for tuning the deformability as compared to crystalline microstructure, and the strength of NPN membranes could be mildly tuned via changing the porosity or pore separation ratio. The possibility of improving the deformability or controlling the strength of NPN membranes by tuning their microstructure is promising and provides insightful guidelines for production of the future generation of NPN membranes. It can also provide a physics-based guideline for tuning the performance of other 2D membranes upon imposing complementary modifications. In this paper, our objective is to design NPN membranes with improved or prescribed strength values. In comparison with our earlier designs^13–15^, our new designs will better resemble the microstructure of experimental NPN membranes. We should note that although one clear path to increase the strength of NPN membrane is to simply decrease their porosity, our focus is to increase their strength at a fixed porosity. The reason behind is that the permeability of NPN membranes decreases with the decrease of porosity^1^ which is not often favorable in the biomedical filtration applications.

**Fig. 1:**
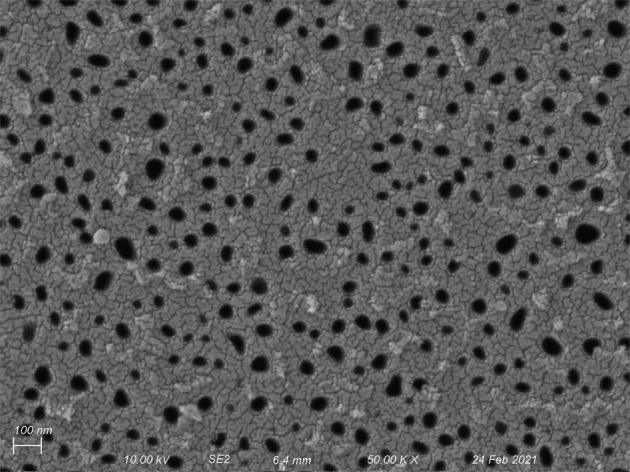
SEM image of the microstructure of NPN sample. Porosity and average pore diameter of the sample are calculated as φ = 11.7% and $\bar{{\rm{D}}}$ = 44.82 nm respectively, and the image was presented in our earlier work^13^.

The microstructure of an experimental NPN sample under scanning electron microscope (SEM) is shown in Fig. 1. As is clear from the figure, the pores in the microstructure of NPN membranes are mostly elliptical with different morphologies (size, aspect ratio, and orientation) and pore density is dissimilar at different regions.

As a result, in this work we aim to consider elliptical pores with different morphologies (size, aspect ratio, and orientation) as well as dissimilar pore density at different regions in our design process. It is shown that mechanical properties of other nanoporous materials such as graphene and hexanitrostilbene are dependent on morphological properties including pore aspect ratio, orientation etc.^16–18^. This implies that those morphological parameters possibly play a role in determining the mechanical behavior of NPN membranes as well. Nevertheless, including all those morphological parameters into the design process of the NPN membranes with desired mechanical properties requires many simulations. This is prohibitively challenging due to the intensive computational time and resources needed to deal with the sophisticated combination of various microstructural parameters.

Machine learning (ML) models are alternative promising tools that can explore the design space in a significantly faster pace in comparison with performing massive number of MD simulations to conduct the forward design approach. Different ML modes such as: support vector machine (SVM)^19^, random forest^20,21^, convolutional neural network (CNN)^22–26^, multi-layer perceptron (MLP) neural network^27^, attention-based transformer neural network^28^ and graph-based neural networks^29^ are adopted as surrogate forward models to relate the microstructures or microstructural features into mechanical properties in many applications. For instance, Yang et al.^22^ combined principal component analysis (PCA) and CNN to predict the stress-strain behavior of binary composites up to the failure point. In a different study, Liu et al.^27^ trained several models including a MLP neural network model based on the results of MD simulations to predict the Young’s modulus and tensile strength of graphene-reinforced nanocomposites. Based on the results of their well-trained model, the authors further modified the micromechanics-based Halpin-Tsai model^30^ which is a widely used model for predicting the elastic modulus of graphene-reinforced nanocomposites. Most recently, Yang and Buehler^31,32^ used graph neural networks (GNN) to predict global properties as well as local behaviors of porous graphene membranes such as atomic stress, and further designed de novo atomic structures with optimum global properties.

Autoencoder (AE), variational autoencoder (VAE), generative adversarial network (GAN), and conditional generative adversarial neural network (cGAN) are another types of ML models that are widely used to design materials^33–39^. For instance, Mao et al.^35^ used GAN to design architectured materials with elastic stiffness corresponding to the Hashin-Shtrikman upper bounds for a range of porosities. In another study, Shen and Buehler^38^ used StyleGAN combined with genetic algorithm to design architected materials with optimized effective modulus. Importantly, the performance of these generative models in designing materials with mechanical properties that are close to the properties of the training dataset is promising^40^. However, it is more desirable to design materials with maximum possible mechanical properties wherein those values are oftentimes outside of the range of the mechanical properties of the training dataset. In this case, further modifications are often needed so that those models can design materials with improved properties that are outside of the range of the properties of the training dataset^35–41^. Examples of such modifications include considering a large enough training dataset that covers the entire design space, or gradually augmenting the small initial training dataset to the region that contains the optimal design while retraining the network simultaneously.

In this work, we implement a deep learning framework that combines GAN and CNN to design NPN membranes with improved or prescribed strength values. In the new designs of NPN membranes, all the pores are elliptical with different morphologies (size, aspect ratio, and orientation) and the pore density is dissimilar at different regions. The NPN membrane with improved strength is called ‘optimized design’ through the rest of this paper. The reason for combining the GAN and CNN in this work is that the GAN component can generate many NPN membranes in an efficient manner and thus accelerates the sampling of the excessively large design space while the CNN component acts as the surrogate model that can quickly evaluate the strength of the membranes. As a result, the CNN component of our framework guides the GAN component to design NPN membranes with our desired strength. It should be noted that the combined GAN-CNN framework was used in several earlier works for property optimization or image diagnostics^33,41–47^. In comparison to earlier works that used GAN-CNN framework, we further add a physics-based interpretation step in the current work which helps to justify the output of our machine learning model based on physics-based simulations. Furthermore, our dataset generation framework can quickly generate many labeled datapoints resembling most of the features of NPN experimental samples, and can be applied easily to investigate other problems in materials science community. As for the training process, we prepare a large dataset of NPN membranes and label them with strength using FE simulation which is computationally more efficient and robust in labeling large dataset as compared to MD simulations. We then train the two components of our deep learning framework based on the labeled dataset wherein the CNN learns to predict the strength of the input NPN image, while the generator of the GAN learns to create generated NPN images from an input random noise vector. Once trained successfully, we use our deep learning framework to design NPN membranes with improved or prescribed strength values. In addition, we compare the strength of the optimized design of the NPN membrane with the strength of common pore patterns such as: cubic and hexagonal pattern. In final, we cross-validate the outcome of our framework by comparing the stress-strain curve of the optimized design with those common pore patterns using MD simulations.

The remaining parts of the paper are organized as follows: The results of our framework including the training process, the designs corresponding with improved or prescribed strength values, as well as the comparison of the optimized design with common pore patterns are discussed in the results section. The discussion section summarizes the work with key conclusions. Finally, the methods section discusses the details of the deep learning framework including the data generation and labeling as well as the setups of our FE and MD simulations.

## Results

### Validation of framework accuracy

**Fig. 2:**
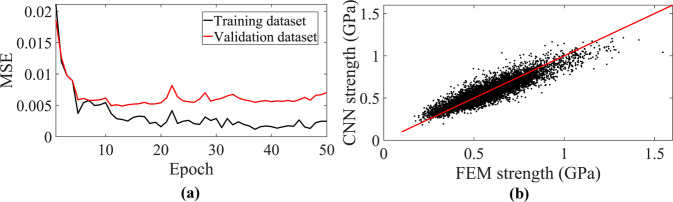
Performance of the CNN component of the deep learning framework. **a** Learning curve of the CNN component during the training process. **b** Parity plot of the testing dataset at epoch = 13 wherein the red line is the reference line.

As it is discussed in the methods section, the two components of our deep learning framework were to be first trained separately before evaluating the performance of our deep learning framework in designing NPN membranes with improved or prescribed strength values. In the first step, we train the CNN component of our deep learning framework on the training dataset for several epochs with batch size of 64 while its performance is evaluated on the validation dataset as is explained in the methods section. The learning curve of the training process for 50 epochs is shown in Fig. 2(a). From the figure, it is seen that the loss value of the training dataset defined based on the mean square error (MSE) drops in the first 15 epochs after which it reaches a plateau. Interestingly, the loss value of the validation dataset shows a similar trend with a minimum value at epoch of 13. The early stopping technique^48,49^ is used to verify that the CNN component starts to over-fit during the training process beyond epoch of 13. Our analysis confirms that epoch of 13 is the first epoch wherein the value of the model’s loss on the validation dataset does not improve for ten epochs. This indicates that the CNN has possibly learned the salient features of the labeled NPN images during the training process at this epoch. To better quantify the performance of the CNN component at this epoch, ground-truth strength values of the testing dataset obtained from FE simulations are plotted versus the predicted strength values obtained from the CNN network in Fig. 2(b) as the ‘parity plot’. From the figure, it is seen that the CNN predicts the strength of the NPN membranes with good accuracy as most of the points lie very close to the diagonal y = x line. This indicates that the CNN component has successfully learned the salient features of labeled NPN images during the training process. It is notable that the values of R^2^, MSE, as well as average relative error for the testing dataset are calculated as 0.8201, 0.0049 and 0.0539 respectively. Nevertheless, one should note that while the overall performance of the CNN is encouraging, the accuracy drops for those data points with high strength values (*σ*_*s*_ > 1 GPa) which is clear from Fig. 2(b). Such a drop could be justified based on the strength distribution of the labeled NPN images of the initial dataset. As is clear from Fig. 9(c) in the methods section, the strength distribution is not completely balanced wherein only few of the NPN membranes possess strength values greater than 1 GPa. This implies that the CNN component receives few datasets with high strength value during the training process. Therefore, the CNN learns the salient features of the labeled NPN images with low strength values better than the ones with high strength. It is worthwhile to note that the number of the convolution layers, depth of the layers, number of the fully connected layers, number of the neurons in the fully connected layers and probability of the drop-out technique that are reported in the methods section along with the value of the batch size are all fined-tuned based on the overall performance of the CNN component on the validation dataset.

In the next step, the generator component is trained for several epochs following the steps that are explained in the methods section to generate NPN images that are indistinguishable from the real labeled dataset. It should be underlined that the NPN images that belong to the initial dataset, are referred as ‘labeled images’ while the images that do not belong to the initial dataset and are created by the generator, are referred as ‘generated images’. Here, the dimension of the latent vector and the batch size are chosen as 25 and 64 respectively and are fined-tuned based on the overall performance of the generator.

To evaluate the performance of the generator during the training process, the Fréchet Inception Distance (FID) score method is implemented^50^. FID is a commonly used metric for evaluating the quality of the generated images that the the generator creates. This method uses the activation distributions of the Inception-v3 model^51^ to quantify the differences between labeled and generated images. Activation distribution is the output of the Inception-v3 model from the last pooling layer prior to the final output layer. We use the pre-trained Inception-v3 model available in Keras^52^ to obtain the activation distributions for 10,000 generated NPN images that the generator creates at different epochs. In addition, the activation distributions of 10,000 labeled NPN images randomly chosen from the real labeled dataset are also obtained. The FID score is then calculated as follows:

1 $${\rm{FID}}={\Vert {\boldsymbol{\mu }}_{\mathbf{1}}-{\boldsymbol{\mu }}_{\mathbf{2}}\Vert }^{2}+Tr({{\mathbf{C}}}_{\mathbf{1}}+{{\mathbf{C}}}_{\mathbf{2}}-2\sqrt{{\mathbf{C}}_{\mathbf{1}}{\mathbf{C}}_{\mathbf{2}}})$$

**Fig. 3:**
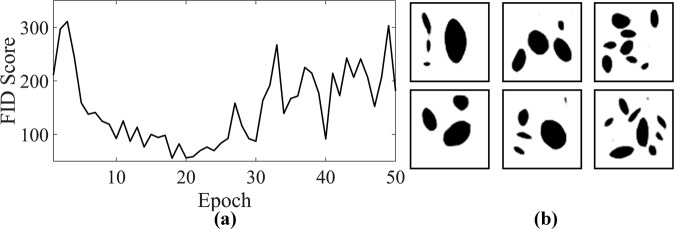
Performance of the generator component of the deep learning framework. **a** The FID score during the training process of the generator component at various epochs. **b** Representative examples of the generated NPN images that are created via the trained generator at epoch = 18.

Where **μ**_**1**_ and **μ**_**2**_ are the mean of the feature vector prepared from the activation distribution for the labeled and generated images, and **C**_**1**_ as well as **C**_**2**_ are the covariance matrix prepared from the activation distribution for the labeled and generated images. The lower the FID score, the better the quality of the generated NPN images from the generator. The FID score is calculated for 50 epochs and is shown in Fig. 3(a). From the figure, it is seen that the generator leads to lowest FID score at epoch 18. To further visualize the performance of the generator at this epoch, several representative examples of the generated NPN images that the generator creates, are shown in Fig. 3(b). Interestingly, it is seen from the figure that the pores in the generated NPN images are elliptical with different morphologies and are distributed heterogeneously. In addition, there is no overlapping between the pores, as well as between the pores and the edges of the membranes. This thus implies that the trained generator successfully creates generated images that encompass the salient features of the labeled NPN images. It is worthwhile to note that the distribution of porosity and pore number of 40,000 generated NPN images are shown in Supplementary Fig. 3 and is further compared with the distribution of the porosity and pore number of labeled NPN images to confirm that they are statistically comparable and thus the generator is well-trained. Also, one of the major challenges of training GAN networks is the occurrence of mode collapse wherein the generator is only capable of generating one or a small subset of distinct images. For the 50 epochs investigated in the current work, mode collapse was not observed based on the latent space size of 25.

### Design performance for prescribed strength values

**Fig. 4:**
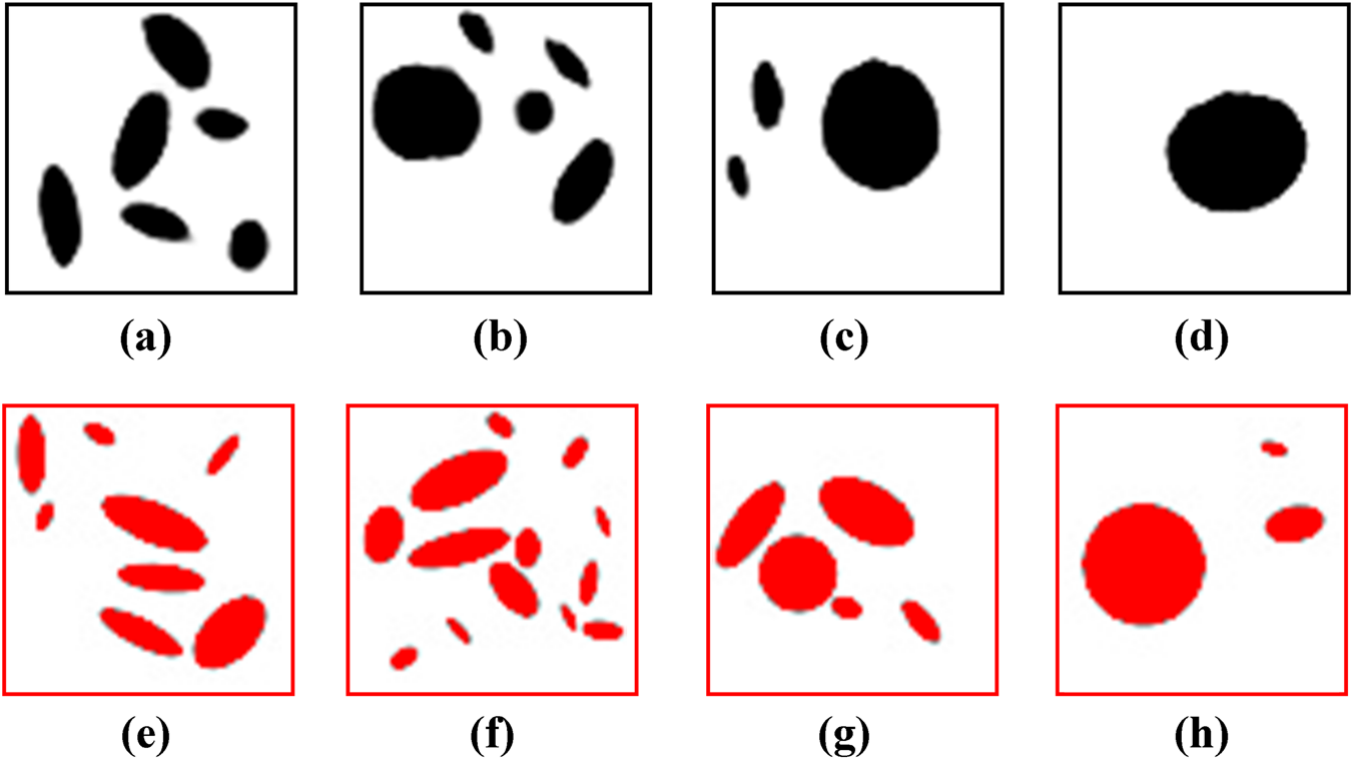
Evaluation of the deep learning framework in designing NPN membranes for prescribed strength. **a**–**d** Four generated NPN images corresponding to strength of 0.4, 0.7, 1 and 1.3 GPa. **e**–**h** Four labeled NPN images from the initial dataset that are comparable with generated images of (**a**–**d**) in terms of strength.

The two components of our deep learning framework are trained successfully as it was demonstrated in the earlier section. Toward investigating the performance of the framework, we couple those two well-trained components and aim to first evaluate its performance in designing microstructures of NPN membranes for prescribed strength values. Designing materials for prescribed properties is important for various operating conditions. For instance, designing porous membranes with prescribed filtration rate is desirable in filtration applications. We thus choose four strength values of 0.4 GPa, 0.7 GPa, 1 GPa, and 1.3 GPa that are all within the strength range of our initial dataset and execute the deep learning framework following the steps explained in the methods section to design NPN membranes for prescribed strength values. The final generated NPN images that the deep learning framework creates for each of the input strength values are shown in Fig. 4(a–d). From the figure, it is seen that the microstructure of the new NPN membranes possesses a combination of circular and elliptical pores with different morphologies depending on the prescribed value of the strength. We recall that the microstructure of experimental membranes also possesses elliptical pores with different morphologies and the pore density is dissimilar at different regions. This thus confirms that our framework successfully designed NPN membranes for prescribed strength values that are closely correlated with the experimental membranes.

We then calculate the porosity of the generated NPN images for the prescribed strength values. For the microstructures shown in Fig. 4(a–d), we obtain the porosity value of 0.194, 0.190, 0.187, and 0.174 respectively. As is discussed in the Supplementary dataset generation, the range of porosity for our initial dataset is (0.1709–0.1842). This implies that while our deep learning framework is only trained on labeled NPN images porosity range of (0.1709–0.1842), once trained, it creates generated NPN images with comparable strength to the labeled data but at even higher porosity. Since strength is traditionally lowered with the increase of porosity, such designs would be useful in designing NPN membranes with improved strength at higher porosity values.

We further explore the initial training dataset to find the labeled NPN images that share similar strength with the four generated NPN images shown in Fig. 4(a–d). Upon comparing the generated NPN images of Fig. 4(a–d) with their correspondent labeled images in Fig. 4(e–h), it is seen that the microstructures of the generated NPN images are completely different from those labeled NPN images of the initial dataset. As a result, our framework learns to not only create generated NPN images with realistic microstructure for prescribed strength values, but also the generated microstructures are distinct from those labeled NPN images of the initial training dataset.

### Design performance for improved strength

**Fig. 5:**
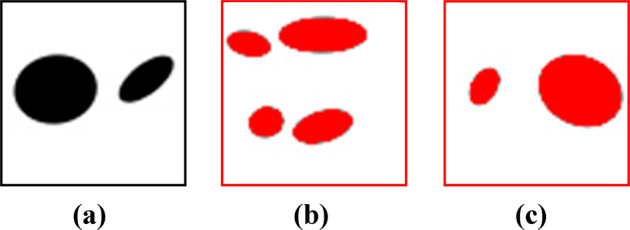
Evaluation of the deep learning framework in designing NPN membranes for improved strength. **a** The generated NPN image corresponding to optimized strength. **b**,**c** NPN images from the initial labeled datasets with strength higher than 1.5 GPa.

Another goal of this work is to design the microstructure of NPN membranes for improved strength which is expected to be higher than the strength values of most of the labeled NPN membranes of the initial dataset. Here, we thus evaluate the performance of the proposed deep learning framework to conduct this task following the steps explained in methods section. The final generated NPN image that the deep learning framework creates for improved strength value is exhibited in Fig. 5(a). This optimized design with porosity of *ϕ* = 0.189 includes one semi-circular and one elliptical pore in which the CNN component of our framework predicts its strength as 1.58 GPa. We recall from the methods section that the highest strength of our labeled NPN images was also 1.58 GPa wherein the porosity of this labeled NPN image, shown in Fig. 5(b), is *ϕ* = 0.176 and it includes four pores. As a result, this indicates that our deep learning framework learns to create generated NPN image with optimized design wherein its strength is comparable with the strength of the labeled NPN image with highest strength in the initial dataset. More importantly, such optimized design possesses a lower number of pores and a slightly higher porosity in comparison with the labeled NPN image with highest strength. This implies that while the strength is generally proportional with the porosity in an inverse manner, it is possible to improve the strength of NPN membranes at a fixed porosity by properly engineering their local pore morphology such as pore pattern, shape etc. As for our optimized design, the strength improvement is related to the local decrease of stress at the stress concentration points as it will be explained in the next section. Similar conclusion was reported based on the MD simulations in our earlier work^13^ as well.

One interesting note here is that in our initial labeled dataset, there are only two labeled NPN images with higher strength than 1.5 GPa. Those labeled NPN images are shown in Fig. 5(b,c) wherein their strength values are 1.58 GPa and 1.53 GPa respectively. This implies that during the training process of our framework, the generator and CNN components received a negligible number of labeled NPN images with higher strength than 1.5 GPa. Upon training, the framework interestingly learns to create new generated NPN image with optimized design that not only possesses higher strength than 1.5 GPa, but also the microstructure of this generated NPN image is distinct from those two labeled NPN images with higher strength than 1.5 GPa.

To further cross-validate the predicted value of the strength of our optimized design from the deep learning framework, we then use FE simulation to compute the strength of the optimized design. From our FE simulation, the strength of the optimized design is obtained as 1.56 GPa which agrees with the value of 1.58 GPa that is predicted from the deep learning framework. The obtained value of strength from FE simulation clearly confirms that our deep learning framework can produce reliable and robust generated NPN images with improved strength comparing with the labeled NPN images of the initial dataset.

### Comparison with common pore patterns

**Fig. 6:**
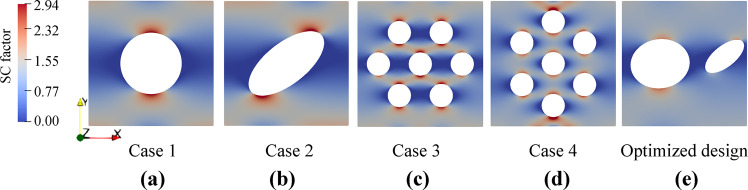
Physics-based interpretation of the optimized design of the deep learning framework. **a**–**e** Distribution of stress concentration (SC) factor of our optimized design along with case 1–4 of common pore patterns calculated with FE simulations. The details of FE simulations were discussed in the Supplementary dataset generation.

Porous membranes with ordered pore patterns such as: cubic and hexagonal patterns are employed widely in different biomedical applications such as: smart filters for bioanalytical devices, plasmonic sensing, cryoEM, flexible electronics, etc.^53,54^. In the microstructure of those porous membranes, all the pores share similar morphology, and the pore density is similar at different regions which is different than the microstructure of the generated or labeled NPN images of our current work. Here, we compare the performance of our optimized design of NPN membrane with the supercell of those conventional membranes to understand the effect of microstructural heterogeneity on the strength of the NPN membranes. Such fundamental understanding provides clear insights to produce the future generations of NPN membranes. The supercells of following four cases of NPN membranes with similar porosity and membrane size as our optimized design are thus constructed and further stretched using FE simulations: (1) Case 1 with cubic pattern and circular pore shape, (2) Case 2 with cubic pattern and elliptical pore shape. The morphology of the elliptical pore including the aspect ratio and orientation is chosen to be consistent with the morphology of the elliptical pore of our optimized design of NPN membranes, (3) Case 3 with hexagonal pore pattern and pore separation ratio of 3.36. It should be noted that case 3 is comparable with the first case of our MD simulations in the earlier work^13^ which was shown to possess highest strength among different patterned membranes. (4) Case 4 with hexagonal pore pattern and pore separation ratio of 0.3. It should be noted that case 4 is comparable with the third case of our MD simulations in the earlier work^13^ which was shown to possess second highest strength among different patterned membranes. The strength of those four cases is calculated from FE simulations as: 1.18 GPa, 1.06 GPa, 1.04 GPa, and 0.83 GPa respectively. The strength of our optimized design from FE simulation is 1.56 GPa. Interestingly, this corresponds with 32% increase in the strength of NPN membranes as compared to the conventional membranes with cubic pore pattern and circular pore. This clearly implies that our optimized design of NPN membrane exhibits higher strength as compared to the common pore patterns. As a result, the microstructural heterogeneity in our optimized design contributes positively to the performance of the NPN membranes. We further exhibit the stress concentration contour of those four cases at small strain in Fig. 6(a–d) to explore the mechanism of the strength improvement of NPN membranes. Upon comparing those stress concentration contours with the stress concentration contour of our optimized design that is shown in Fig. 6(e), it is seen that the stress concentration factor at the stress concentration points is lower for our optimized design. Specifically, the maximum stress concentration factor of the four cases is obtained as 3.12, 3.49, 3.56, and 4.50 while the corresponding value for the optimized design is 2.39. The stress concentration factor is thus lowest for our optimized design in comparison with all four cases. As a result, the optimized design is expected to reach a higher strength in comparison to common pore patterns. This indicates that the microstructural heterogeneity of our optimized NPN design facilitates the stress distribution inside the NPN membrane and lowers the stress concentration in comparison with microstructural homogeneity of the common pore pattern.

**Fig. 7:**
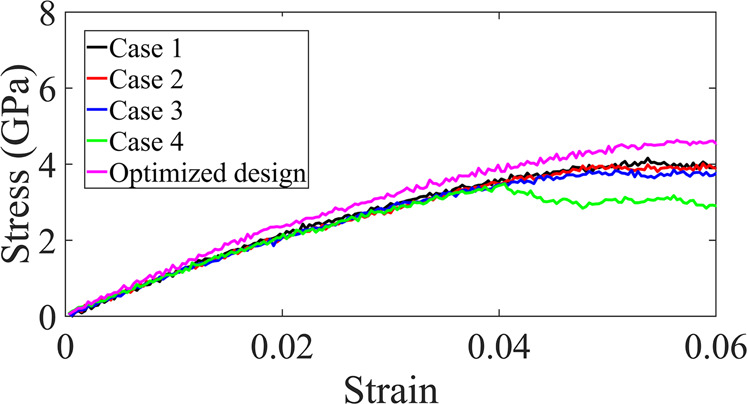
Cross-validation of the optimized design of the deep learning framework with MD simulations. Stress-strain curves of the NPN membranes with different common pore patterns as well as the generated NPN image (optimized design) with improved strength obtained from MD simulations.

In the final section, we use MD simulations to mimic the uniaxial tensile loading on the five NPN membranes and further cross-validate the performance of our optimized design in comparison with the investigated common pore patterns. The stress-strain curves of the samples up to 6% of strain are thus shown in Fig. 7. From the figure, it is seen that the stress-strain behavior of our optimized design outperforms the behavior of common pore patterns. MD simulations thus provide additional support for the strength superiority of our optimized design as compared to the common pore patterns. As a result, the deep learning framework presented in this study shows not only a good accuracy in terms of predicting the strength of NPN membranes with different microstructures, but also offers avenues to generate optimized design with improved strength that outperforms conventional membranes with common pore patterns.

## Discussion

NPN membranes are amorphous brittle materials with growing applications that are not well-studied yet. The mechanical properties of the current generation of these membranes need to be improved for further extending their biomedical application in life-saving areas such as: wearable hemodialysis devices. Computational design of NPN microstructures with improved strength that are closely correlated with the microstructures of experimental samples is a challenging task owing to the complexities of their microstructure. We proposed a deep learning framework to design NPN membranes for optimized or prescribed strength values. The framework employed a deep learning design approach combining the generator of GAN to generate NPN images and a deep CNN to predict the strength of the generated NPN images. Below are the conclusive highlights of our work:

- The deep learning framework learned to not only generate NPN images with realistic microstructure for prescribed strength values, but also it generated NPN images distinct from the labeled NPN images of the initial training dataset. In addition, our framework was able to design an optimized NPN membrane with a heterogeneous microstructure and strength higher than almost all the labeled NPN images of the initial dataset.
- We added an interpretation step to our framework and justified the output of our deep learning model using physics-based simulations. Excellent agreement between the strength value of our optimized design predicted from the deep learning framework and FE simulation confirms that the framework was able to generate reliable and robust NPN image with improved strength comparing with the initial dataset. Using FE simulations, we compared the strength of our optimized design with the supercell of conventional membranes that possess homogenous microstructure. Our results unveiled that the optimized design outperforms the conventional membranes. Our FE simulations revealed the reasoning behind this key finding and showed that the microstructural heterogeneity of the optimized design, lowers the stress at the concentration points around the pores which results into significantly increase of the strength. As a result, our framework was able to design NPN membranes with improved strength, while the physics-based interpretation showed that the deep learning framework correctly learned that microstructural heterogeneity is a key for the optimized design.

- Apart from FE simulations, the strength superiority of our optimized design was cross-validated with MD simulations as well. Although our framework is trained based on the linear elastic behavior, our MD simulations confirmed that our optimized design performs better than the common pore patterns beyond the elastic regime as well.
- The framework we developed in this work can easily be applied and generalized to design other materials for different applications. For instance, in a separate work we have been currently utilizing the framework to design materials that can survive in the environments that involve severe irradiation such as nuclear fusion and fission reactors. The advantages of using CNN alongside a generator are: (1) we only need a limited amount of sample space for training the two components of our deep learning framework. (2) The framework can provide a quick and brand-new optimized design for the inverse design problem. Our data-driven model suggests practical solutions to materials design using computationally inexpensive models as compared to the expensive physics-based simulations, and could significantly save the time and cost to discover new materials.

## Methods

### Deep learning framework overview

**Fig. 8:**
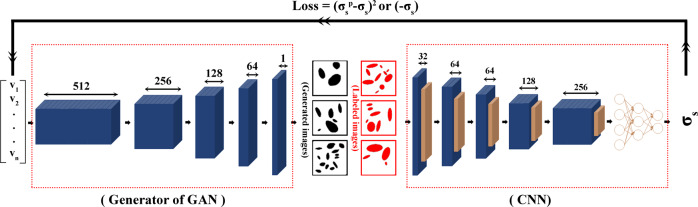
Schematic of the architecture of the deep learning framework. This architecture combines the generator of GAN^59^ and a deep CNN^55^ wherein the convolutional layers, max-pooling layers, and neurons are shown with blue, brown and circles respectively. The depth of the convolution filters is mentioned over the double arrows.

Toward the goal of this work in designing NPN membranes for improved or prescribed strength values, we need to design a framework that can create thousands of NPN microstructures and evaluate their strength performance in a fast pace. The framework is required to be able to: (1) predict the strength of NPN membranes based on their microstructures, (2) generate NPN microstructures that encompass elliptical pores with different morphologies and dissimilar pore density at different regions, (3) connect the earlier two tasks to design NPN membranes for improved or prescribed strength values. For the first purpose, we choose to add a CNN network into our framework which is known to be good at predicting the output properties from input images upon learning the salient features of the images during the training process. The architecture of the CNN network in our deep learning framework highlighted in Fig. 8 is adopted from the work of LeCun et al.^55^ and is further fine-tuned for predicting the strength of NPN membranes. We choose this architecture due to its great performance as is reported in the earlier works^56–58^ along with its simplicity. As for the second purpose, we choose to add a generator network of GAN into our framework which is known to be good at generating images from input noise vector wherein the generated images encompass the salient features of the real labeled images of the training dataset. The architecture of the generator in our framework is adopted from the work of Radford et al.^59^ and is highlighted in Fig. 8. As for the third purpose, we couple the two network and further minimize the gradient of the following loss functions with respect to the components of the latent vector:

2 $${{Loss}}={({\sigma }_{s}^{P}-{\sigma }_{s})}^{2}$$

3 $${{Loss}}=-{\sigma }_{s}$$

Where ${\sigma }_{s}^{P}$ is the prescribed strength and *σ*_*s*_ is the predicted strength from CNN network. The obtained latent vector is then used as an input for the generator to create the microstructure of the NPN membrane corresponding to the prescribed or improved strength values. It is noted that our deep learning framework is implemented in Tensorflow^60^.

### Deep learning framework development

The following steps are carried out to develop our framework:

#### Dataset preparation

**Fig. 9:**
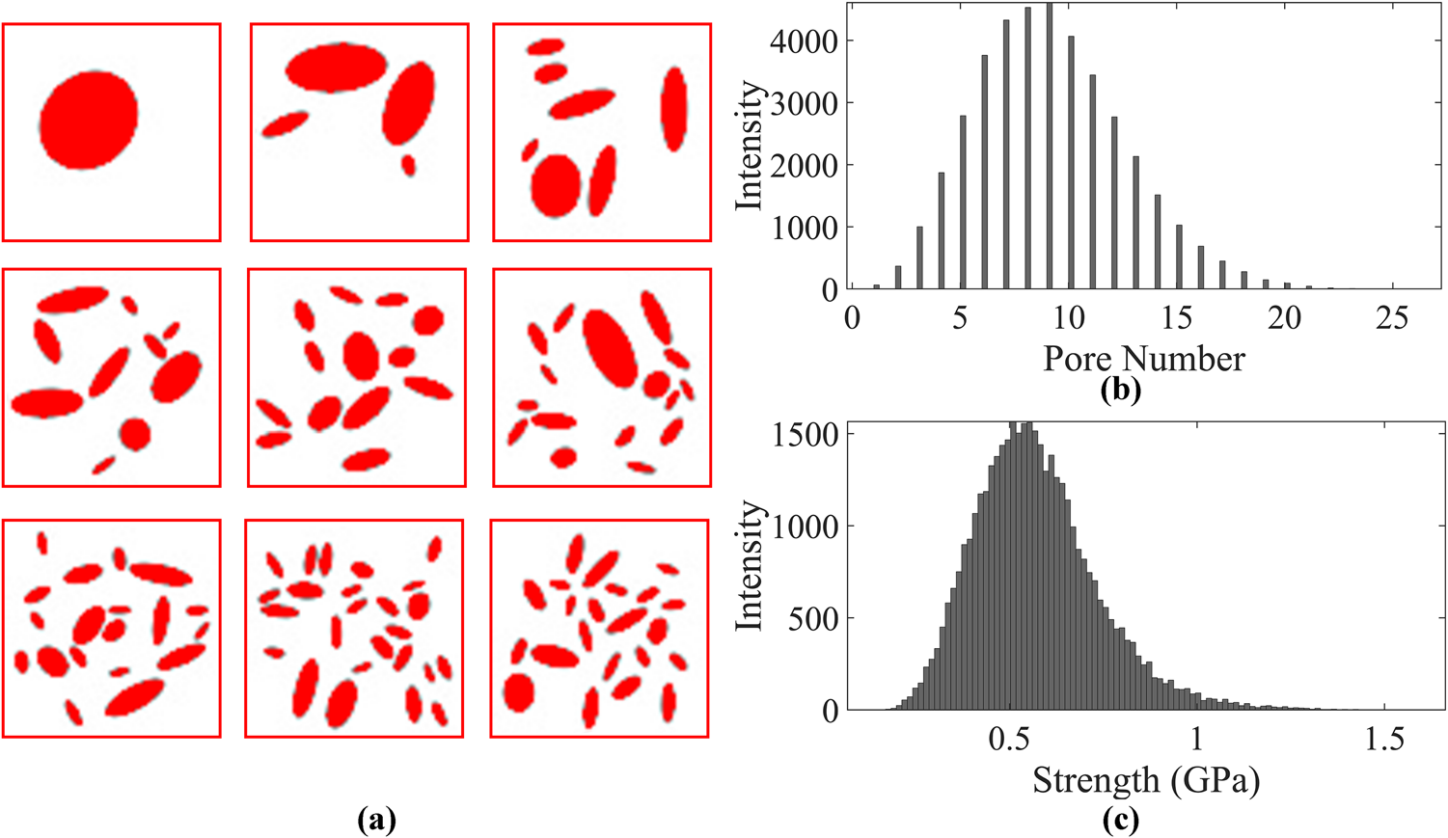
Visualization of the initial dataset obtained from the dataset generation step. **a** Representative examples of the labeled images from the initial NPN dataset. **b** Distribution of pore number for the initial NPN dataset. **c** Distribution of strength for the initial NPN dataset.

The first step in developing our deep learning framework is to prepare a dataset that is labeled with strength. For that, a similar method that is proposed by Wang et al.^61,62^ is used to generate a dataset of 40,000 grayscale images with 64 × 64 × 1 pixel shown in Fig. 9(a). The average porosity of the dataset is 0.177 and the range of the pore numbers is 1 < N < 25, shown in Fig. 9(b), that are both comparable with the MD simulations of our earlier paper^13^. In this paper, all the NPN images from this dataset will be presented with red color and will be referenced as ‘labeled NPN image’ to avoid any confusion with generated NPN images from the generator component of our framework that are colored in black. The NPN membranes of the initial dataset are then labeled with their corresponding strength values via carrying out FE simulations using open-source Python packages SfePy^63^ to conduct the FE simulations and GMSH^64^ to mesh the NPN microstructures. Details of the dataset generation and their labeling process are discussed in the Supplementary dataset generation. Briefly, linear elasticity is assumed in the FE simulations and the inclusion of nonlinear elasticity, plasticity, and crack propagation are left for future studies. In addition, plane stress assumption is imposed on the FE simulations to mimic the thinness of NPN membranes. To simulate the uniaxial loading, the displacement of all the left edge nodes of the NPN membranes, such as the one shown in Supplementary Fig. 2(b), is constrained along x while all the degrees of freedom (DOF) of the central node on the left edge is constrained. We then impose a uniform displacement field on the right edge nodes. Once the local normal strain of the element with highest strain value, i.e., critical element which is near the pores, reaches the failure value, the strength is calculated via following equation^65,66^:

4 $${\sigma }_{s}=\frac{1}{N}\mathop{\sum }\limits_{i=1}^{N}{\sigma }_{i}$$

Where σ_i_ is the stress of the ith element and N is the number of total elements. The strength distribution for all 40,000 NPN membranes of the initial dataset is shown in Fig. 9(c). This distribution resembles a Gaussian distribution with a mean value of 0.54 GPa. It is notable that while the lower-end and higher-end tails reach 0.14 GPa and 1.58 GPa respectively, the number of data points around the tails are negligible. Specifically, there are only two NPN membranes in which their strength exceeds 1.5 GPa.

#### CNN training

The architecture of the CNN network, i.e., the predictive component of our deep learning framework, is shown in Fig. 8 which contains five convolution layers and two fully connected layers with 128 and 32 neurons respectively. The rectified linear unit (ReLU) activation function which is followed with a max-pooling layer is applied on all convolution layers, and a filter size of 3 × 3 is used. In addition, drop-out technique^67^ with probability of 0.5 is imposed on the final convolution layer as well as first fully connected layer to prevent the overfitting in the training process. 80% of the real labeled dataset is randomly chosen as the training dataset while the rest 20% is used as the validation dataset which helps to detect the overfitting and perform model selection during the learning process. In addition, a separate dataset of 8000 labeled NPN images is prepared as the testing dataset to examine the performance of the final well-trained model. In this approach, the CNN learns to predict the strength of the NPN images via minimizing the mean square error (MSE) loss function through backpropagation. In this work, the architecture of the LeNet CNN model^55^ is used and further fine-tuned for predicting the strength of NPN membranes as is mentioned in the earlier part of the methods section.

#### Generator training

The generator network, i.e., the generative component of our deep learning framework, generates NPN microstructures from the input random noise vector with bounded elements between (−1, 1). The architecture of the generator component is shown in Fig. 8 which is based on deep convolutional generative adversarial network (DCGAN) that is proposed by Radford et al.^59^ as is mentioned in the earlier part of the methods section. Briefly, the latent vector is first projected and reshaped into a feature map with 4 × 4 × 512 dimensions which is then fed into four fractionally-strided convolution layers, and the output is 64 × 64 × 1 pixel image. To accelerate the training speed of feature extraction, batch normalization is applied to all layers, and a filter size of 5 × 5 is used. The rectified linear unit (ReLU) is utilized as the activation function of the first four layers while Tanh is imposed on the final layer. To train the generator, it is coupled with another network known as discriminator and both networks are trained adversarially via minimizing the binary cross entropy loss function through backpropagation^68^. In this approach, the generator learns to generate NPN images that are indistinguishable from the real labeled dataset, while the discriminator learns to classify the labeled images from the generated NPN images simultaneously. The architecture of the discriminator is comparable with the generator and contains four convolution layers. However, it is fed by 64 × 64 × 1 pixel images and results in one-dimensional output to classify the generated NPN images from the labeled ones.

#### Inverse design process

After training the CNN and generator networks separately, we combine the two networks to complete our deep learning framework. To design the microstructure of the NPN membranes for improved or prescribed strength values, the gradient of the loss functions defined as Eq. (2) and Eq. (3) should be minimized with respect to the components of the latent vector respectively using Limited-memory Broyden–Fletcher–Goldfarb–Shanno algorithm (L-BFGS) method^69^. Upon successful minimization of the loss function, the obtained latent vector will be then used as an input for the generator to create the corresponding microstructure of the NPN membrane.

### MD simulations

MD simulations are conducted using the open-source program package Large-scale Atomic/Molecular Massively Parallel Simulator (LAMMPS)^70^. 3-body Vashishta interatomic potential^71^ is chosen from the available interatomic potentials to model the mechanical behavior of amorphous NPN membranes. The ability of this potential to realistically mimic the mechanical behavior of amorphous silicon nitride structures has been verified in several studies before^71–73^. Periodic boundary conditions are applied along in-plane directions while free boundary condition is applied in the Z direction to mimic the thin thickness of the NPN membranes along z direction. Amorphous NPN membranes are prepared following the approach that is explained with detail in our earlier work^13^. Prepared NPN membranes are then relaxed to their minima following a 100 picosecond equilibration in an NPT ensemble (i.e., constant number of particles, constant pressure, and constant temperature) equilibration at 300 K^74^. The tensile test is performed through NPT ensemble with a strain rate of 5 × 10^8^ s^−1^.

## Supplementary information

SUPPLEMENTAL MATERIAL

## Data Availability

The data used and/or analyzed during the current study are available from the authors upon request.

## Code availability

The codes developed in this study are available from the authors upon request. To implement the GAN-CNN component of our deep learning framework, the codes of Tan et al.^33^ that are publicly available at https://github.com/hkust-ye/rktan-networkInverseDesign were utilized, and were further modified.
