# Supplementary material for: An interpretable deep learning approach for designing nanoporous silicon nitride membranes with tunable mechanical properties
Source: NPJ Comput Mater. 2023 May 27;9(1):82. doi: 10.1038/s41524-023-01037-0 (PMC10221757; doi:10.1038/s41524-023-01037-0)
Supplement: Supplementary file 1 — SUPPLEMENTAL MATERIAL [file 41524_2023_1037_MOESM1_ESM.pdf]

Supplementary material for

An interpretable deep learning approach for designing nanoporous silicon nitride membranes with tunable mechanical properties

Ali K. Shargh[1] and Niaz Abdolrahim[1, 2, 3, *]

[1]Department of Mechanical Engineering, University of Rochester, Rochester, New York 14627, United States

[2]Materials Science program, University of Rochester, Rochester, New York 14627, United States

[3]Laboratory for Laser Energetics, University of Rochester, Rochester, New York 14627, United States

*Email: niaz@rochester.edu

Supplementary dataset generation

Here, the idea is to: 1) generate an elliptical pore with random morphology including: pore orientation, aspect ratio, and size shown with θ, AR, and R respectively, 2) choose a random position inside the matrix and examine whether the candidate pore intersects with other pores as well as the membrane edges or not, 3) place the candidate pore in the chosen random position if the pore does not overlap with the remaining pores while return to step 1 if the candidate pore intersects with other pores, 4) repeat earlier steps until the porosity reteaches close to the desired value. In this work, we choose 0.177 as the desired value of porosity which is similar to the average porosity value used in MD simulations of our earlier paper [1]. It is worthwhile to note that the values of the morphological parameters of the pores are randomly chosen from the ranges: $0^o < \theta < 180^o$, $1 < AR < 3.5$ and $3.06 < R < 16.25$. The first two ranges are determined based on our analysis of

the microstructure of 55 experimental NPN membranes that are shown in Supplementary Figure 1.

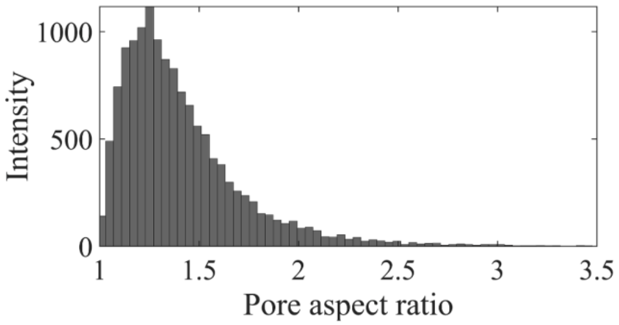

(a) **(b)**

Supplementary Figure 1: Visualization of the 55 experimental samples. Distribution of (a) pore aspect ratio and (b) pore orientation of all the nanopores of 55 experimental NPN membranes that were presented in our earlier work [1]

The third range which is indirectly correlated with the range of the pore number N, is chosen carefully to result in $1 < N < 25$ for our dataset as is shown in Figure 9(b) of the paper. Such range of pore number is chosen to be comparable with the MD simulations of our earlier paper [1]. The distribution of porosity of all 40,000 NPN membranes are depicted in Supplementary Figure 2(a). In addition, a critical minimum distance of 1 pixel is imposed in between the pores, as well as between the pores and the edges of the membranes to avoid any probable overlapping.

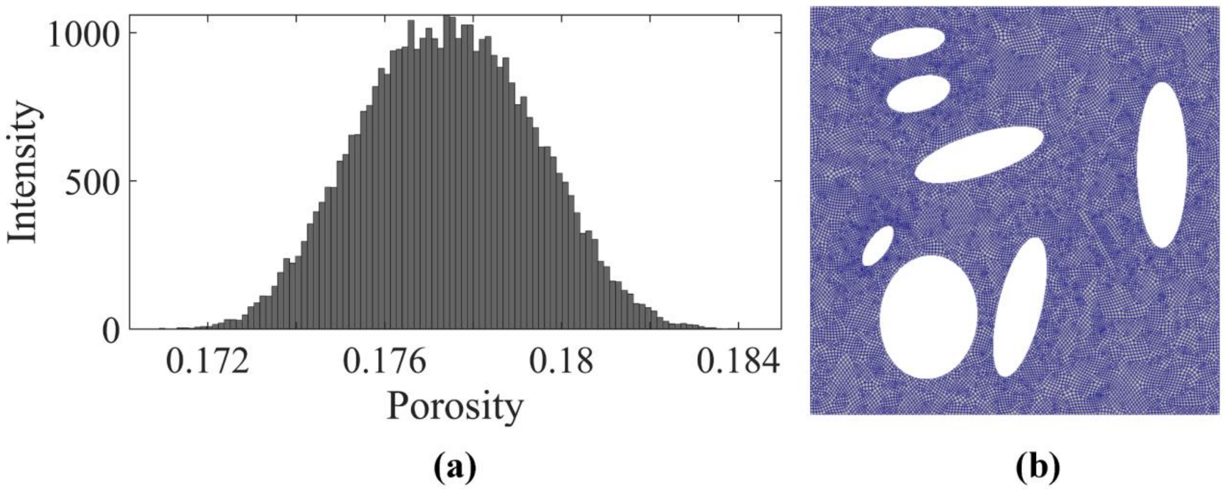

Supplementary Figure 2: Visualization of the initial dataset obtained from the dataset generation step. (a) Distribution of porosity for the initial NPN dataset. (b) Representative example of NPN membrane with discretized domain that is visualized via open-source PovRay.

We then label the NPN membranes with their strength. To do so, the NPN membranes are first discretized by 4-node square elements. The Young's modulus, Poisson ratio and failure strain of the silicon nitride are set to 185.2 GPa, 0.21 and 0.02 respectively that are required to calibrate our FE model for simulating the mechanical behavior of NPN membranes in an accurate manner. These values are all based on our MD simulations of non-porous silicon nitride membrane under tensile loading from our earlier paper [1]. Briefly, the Young's modulus is calculated from the linear part of the stress-strain curve, failure strain is calculated from the stress-strain curve using the 0.2% standard offset method and the Poisson ratio is calculated based on the strain values under uniaxial tensile loading. To tackle the mesh convergence issue of the strength calculation in an automatic manner, we repeat the strength calculation from Eq. (4) of the paper based on the nodal values as follows: upon imposing a uniform displacement field on the right edge nodes, once the strain of the node with highest strain value, i.e. critical node, reaches the failure value, the strength is calculated from Eq. (4) of the paper wherein σ_i will be the stress of the ith node and N is the number of total nodes in this new calculations. We decrease the mesh size continuously until the

difference between the strength value that is calculated from nodal values and element values are within 7% of difference.

Supplementary statistical analysis of the generator performance

The distribution of porosity of 40,000 generated images using the well-trained generator is shown in Supplementary Figure 3(a) which is closely analogous with the distribution of the porosity of the initial dataset shown in Supplementary Figure 2(a). Briefly, both distributions resemble a gaussian distribution around mean porosity of 0.18 although the distribution for the initial dataset is narrower than the other one. Specifically, mean (μ) and standard deviation (σ) values of generated datapoints are 0.179 and 0.012 respectively while the corresponding values for initial dataset are 0.177 and 0.001. Similarly, the distribution of the pore number of those generated images shown in Supplementary Figure 3(b) with μ=8.8 and σ=3.2 is comparable with the distribution of the pore number of the initial dataset shown in Figure 9(b) of the paper with μ=9.04 and σ=3.46.

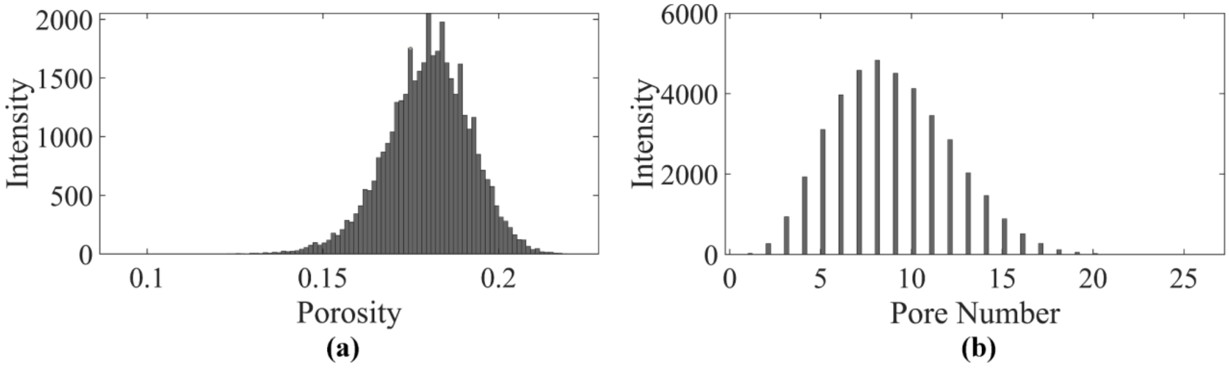

Supplementary Figure 3: Visualization of the dataset obtained from the well-trained generator. Distribution of porosity (a) pore number (b) pore number for 40,000 generated NPN images that are created via trained generator at epoch=18

Supplementary references

1. Shargh, A. K., Madejski, G. R., McGrath, J. L. & Abdolrahim, N. Mechanical properties and deformation mechanisms of amorphous nanoporous silicon nitride membranes via combined atomistic simulations and experiments. *Acta Mater.* **222**, 117451 (2022).
